# Role of FGF-19, FGF-21 and FGF-23 in Fetal and Neonatal Growth

**DOI:** 10.3390/jcm14134520

**Published:** 2025-06-26

**Authors:** Anna Rzewuska-Fijałkowska, Wojciech Kwaśniewski, Tomasz Gęca

**Affiliations:** 1 Department of Obstetrics and Pathology of Pregnancy, Medical University of Lublin, 20-081 Lublin, Poland; tomasz.geca@umlub.pl; 2 Department of Oncological Gynecology and Gynecology, Medical University of Lublin, 20-081 Lublin, Poland; wojciech.kwasniewski@umlub.pl

**Keywords:** FGF-19 subfamily, fetal development, gestational diabetes mellitus (GDM), gestational hypertension, intrauterine growth restriction

## Abstract

**Background:** The Fibroblast Growth Factor (FGF) 19 subfamily plays a key role in the regulation of metabolic and growth processes, and their dysregulation can lead to fetal growth disorders, such as small for gestational age (SGA) and large for gestational age (LGA), as well as to pathogenesis and development of gestational diabetes and gestational hypertension. **Methods:** We conducted a narrative review using the PRISMA2020 statement. Two electronic databases were searched: PubMed and Web of Science until October 2024. The search terms were as follows: (FGF-21 OR fibroblast growth factor-21 OR FGF-23 OR fibroblast growth factor-23 OR FGF-19 OR fibroblast growth factor-19) AND (human fetus development OR fetal growth OR infancy). We only included original papers that analysed the effect of FGF-19,21,23 on pre- and postnatal development. **Results:** Only 6 out of 203 studies met the inclusion criteria. There were higher concentrations of FGF-21 among patients with gestational diabetes mellitus (GDM) compared to healthy females, but no differences were found in FGF-21 values in newborn’s umbilical cord blood. Interestingly, higher FGF-21 concentrations were observed in females than males born to patients with GDM. FGF-19 was linked to fetal development by its association with chronic insulin secretion levels during fetal life, particularly in female newborns, but no significant correlation with GDM was found. The evaluation of the role of FGF-23 has shown that its low level could be related to gestational hypertension and fetal growth restriction. **Conclusions:** In conclusion, all the studies discussed suggest that FGF-19 subfamily factors may play an important role in fetal and neonatal growth and development, particularly in pregnancies complicated by metabolic disorders, such as gestational diabetes or gestational hypertension. Differences in FGF-19 and FGF-21 concentrations based on gender and gestational disorders suggest the need for further research in order to fully understand the effects of these proteins and their potential clinical applications.

## 1. Introduction

One of the key aspects of prenatal care is the monitoring of intrauterine fetal growth by estimating fetus weight using biometric measurements during ultrasound examinations. The values are then plotted on centile growth charts, and the norm for fetal weight is between the 10th and 90th centile for a given gestational age. Fetal growth abnormalities are divided into hypotrophy, which occurs when the estimated fetal weight is below the 10th centile, and hypertrophy, when the predicted weight is above the 90th centile [1,2].

Hypotrophic fetuses can be divided into two groups: constitutionally small fetuses (SGA—small for gestational age), which have a low body weight due to genetic conditions (e.g., short parents), but are growing normally and have normal vascular flow parameters during ultrasound examinations, and fetuses with fetal growth restriction (FGR), which do not reach their growth potential due to detrimental external factors, such as placental insufficiency, mother’s nicotinism, addiction to drugs and psychoactive substances, pre-pregnancy and gestational diabetes mellitus (GDM), hypertension, history of pre-eclampsia, cardiovascular disease, vertical infections (toxoplasmosis, rubella, cytomegaloviral disease, herpes type 2—TORCH) and Coxsackie or chickenpox viruses [1].

In contrast, the term fetal hypertrophy is used interchangeably with ‘large for gestational age’ (LGA), meaning that the predicted fetal weight exceeds the 90th centile. Fetal macrosomia, on the other hand, means that the estimated fetal weight exceeds 4000 g or 4500 g, regardless of gestational age [2].

Fibroblast growth factors (FGFs) play an important role in various biological processes, including the regulation of metabolism and homeostasis. The FGF family comprises 22 proteins that serve different functions depending on the tissues they affect. The FGF-19 subfamily, also known as atypical and endocrine FGFs, includes FGF-19, FGF-21 and FGF-23, which, unlike the other FGF subfamilies, have a reduced affinity for heparin-binding regions, and thus act as hormones [3]. Endocrine FGFs require the presence of the Klotho protein, which acts as a coreceptor on target cells, allowing efficient binding to FGFR receptors [4].

FGF-19 is mainly produced in the ileum and its secretion is stimulated by bile acids in enterohepatic circulation [5]. In addition to bile acid metabolism, FGF-19 plays a significant role in regulating glucose and lipid metabolism. It is also involved in the regulation of energy balance, as it promotes fat oxidation and reduces body weight in animal model studies. In addition, it has been shown to reduce insulin resistance and hepatic steatosis [6].

Whereas FGF-21 plays a key role in regulating glucose and lipid metabolism, it induces glucose uptake in adipocytes, lowers blood glucose levels and increases insulin sensitivity [7]. Moreover, FGF21 inhibits lipolysis and serves a protective function in starvation, promoting the body’s adaptive response through ketogenesis and fatty acid oxidation [8].

FGF-23 is a polypeptide synthesized mainly by osteocytes and osteoblasts, and it mainly affects the kidneys and parathyroid glands [9]. What is more, FGF-23 is also found in skeletal muscle, liver, spleen and heart. It is a key regulator of phosphate–calcium metabolism and its overexpression can lead to hypophosphatemia. In response to elevated phosphate or vitamin D concentrations, FGF-23 reduces phosphate absorption in the kidneys, regulating the body’s mineral balance [5].

The FGF-19 subfamily plays a key role in the regulation of metabolic and growth processes, and their dysregulation can lead to fetal growth disorders, such as SGA and LGA, as well as to pathogenesis and development of GDM and gestational hypertension [10].

The aim of this study is to assess the role of the FGF-19 subfamily in fetal and neonatal growth.

## 2. Materials and Methods

**Figure 1:**
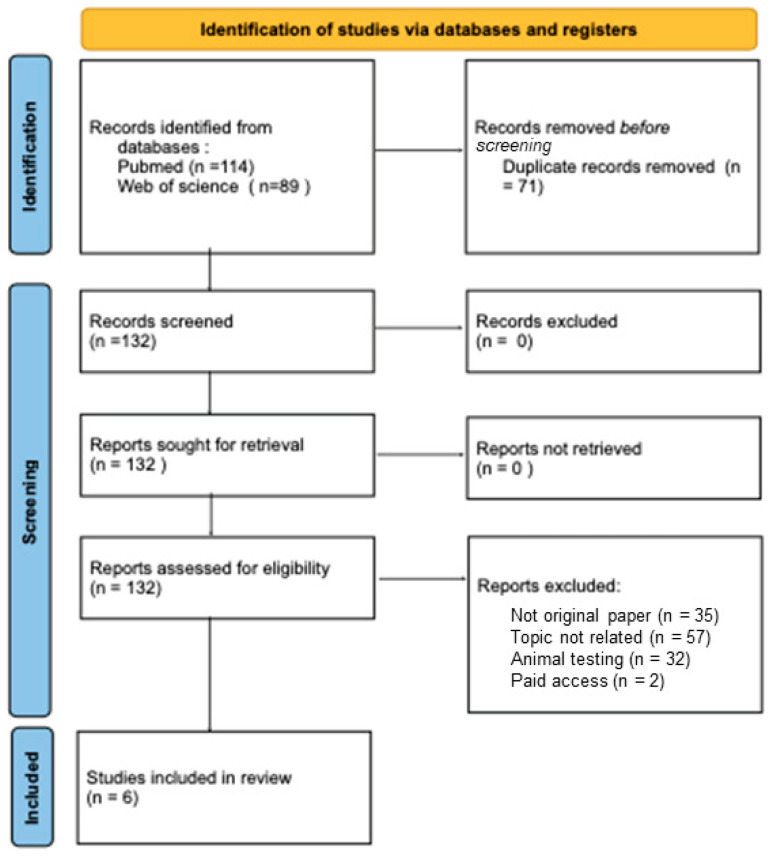
PRISMA diagram (figure self-created using PRISMA pattern [11]).

We conducted a narrative review using the PRISMA2020 statement (Figure 1) [11]. Two electronic databases were searched: PUBMED and https://www.webofscience.com/wos/woscc/basic-search ( accessed on 1 September 2024) until 1 October 2024. The search terms were as follows: ((FGF-21 OR fibroblast growth factor-21 OR FGF-23 OR fibroblast growth factor-23 OR FGF-19 OR fibroblast growth factor-19) AND (human fetus development OR fetal growth OR infancy). Only six studies met the inclusion criteria, which were original papers written in English, studies conducted on a group of humans, no use of animals, and topic concerning the effect of the FGF-19 subfamily on pre- and postnatal development.

## 3. Results

Megia et al. [10] were the first to demonstrate the presence of FGF-21 in umbilical cord blood, investigating its dependence on maternal factors and postnatal growth of newborns. Blood samples were collected from 157 pregnant women, including 79 with GDM and 78 healthy women, and their newborn babies (cord blood). Neonatal birth weight and length were also assessed immediately after birth and at 12, 24 and 48 months of age, and BMI-ZS was calculated on the basis of these measurements. The ELISA test was used to measure FGF-21 levels in the samples. A higher concentration of FGF-21 was observed in the blood of women with GDM compared to healthy pregnant women. However, there were no significant differences in FGF-21 concentrations in cord blood in the children of patients from either group. In contrast, FGF-21 values in cord blood of children of patients with and without GDM correlated positively with maternal blood FGF-21 concentrations and negatively with total cholesterol, HDL and BMI-ZS values at birth. On the other hand, in infants of mothers without GDM, positive correlations were observed between FGF-21 values and BMI ZS at 12, 24 and 48 months of age—*p* < 0.05. Furthermore, among women with GDM treated with insulin or diet alone, there were no statistically significant differences in FGF-21 concentrations in both maternal blood and cord blood. It was also noted that females born to women with GDM had higher cord blood FGF-21 concentrations compared to males [10].

In a study by Sánchez-Infantes et al. [12], changes in blood concentrations of FGF-19 and FGF-21 during early infancy were analysed. The study included 22 neonates with normal weight AGA (appropriate for gestational age) and 22 with low body weight SGA, as well as 35 healthy, non-obese adults. Measurements were taken at 0, 4 and 12 months in infants with AGA, at 0 and 4 months in infants with SGA, and ELISA assays were used to determine FGF-19 and FGF-21 values. FGF-19 and FGF-21 values in females and males with AGA were comparable, so the two groups were combined. It was observed that FGF-19 concentrations in cord blood were low (24.0 ± 6.9 pg/mL) and remained at similar levels throughout the first week of life (32.9 ± 19 pg/mL). On the other hand, after four months, FGF-19 values in AGA groups increased rapidly (614.8 ± 70.7 pg/mL; *p* < 0.0001), significantly exceeding typical adult values (approximately 300 pg/mL). When measured in 12-month-old children, FGF-19 concentrations decreased (314.5 ± 61.8 pg/mL; *p* < 0.001 compared to month 4). FGF-21 concentrations in cord blood at birth were also low (42.8 ± 3.9 pg/mL) but increased significantly during the first week of life and remained at a higher level during the first year (307.1 ± 67.9 pg/mL at month 4 and 448.9 ± 175.6 pg/mL at month 12), reaching values 1.4 times higher than in adults (approximately 150 pg/mL). It was observed that FGF-19 values in SGA neonates (49.8 ± 19.0 pg/mL) were not significantly different from those of AGA infants at birth; however, at 4 months of age, SGA infants had significantly lower FGF-19 concentrations (302.4 ± 50.5 pg/mL) compared to AGA infants (614.8 ± 70.7 pg/mL; *p* < 0.0001), whereas FGF-21 concentrations were comparable in both groups, both at birth and at 4 months of age (43.2 ± 7.9 pg/mL and 260.1 ± 45.0 pg/mL). Furthermore, the mode of delivery did not affect FGF-19 and FGF-21 concentrations [12].

*Increased Fibroblast Growth Factor 21 (FGF-21) Concentration in Early Second Trimester Amniotic Fluid and Its Association with Fetal Growth* is the first study to assess the relationship between FGF-21 and insulin concentrations in amniotic fluid during the early second trimester of pregnancy and estimated fetal weight at the end of the second and third trimester. The study group included 31 SGA fetuses and 18 LGA fetuses, and the control group consisted of 31 AGA fetuses. All study participants were divided according to gestational age-early second trimester (15–22 weeks gestation), fetal sex and maternal height and weight, and ELISA kits were used to measure FGF-21 levels. It was observed that higher FGF-21 concentrations in the amniotic fluid in the early second trimester were associated with abnormal fetal growth, with both SGA and LGA fetuses having higher FGF-21 values compared to AGA fetuses. Furthermore, in SGA fetuses, it was observed that, as the centile values decreased, the blood FGF-21 values gradually decreased; despite this, among SGA fetuses below the 5th centile, the FGF-21 concentration was still higher than in the control group, and only in fetuses below the 3rd centile were the FGF-21 concentration values lower than in the control group (*p* < 0.01). However, a gradual increase in FGF-21 values was observed in 5 LGA fetuses above the 95th percentile. Furthermore, the study found that maternal age, female sex of the fetus and smoker status of the mother were associated with SGA, while this was not observed for LGA. Insulin levels were higher in SGA and LGA fetuses, although the differences were not statistically significant. There was also no statistically significant relationship between maternal age, weight and height and FGF-21 concentrations. The authors suggest that increased levels of FGF-21 may have an adaptive effect, increasing energy availability and protecting against oxidative stress and promoting tissue repair, which may be crucial in cases of FGR [13].

A study by Joung et al. [14] aimed to investigate the relationship between FGF-21 concentrations in the first week of life and weight gain in hospitalised preterm infants. The study included 25 preterm infants born between 24 and 36 weeks, from whom blood samples were taken within the first 24 h of life (T1) and then 24–96 h after the first blood draw (T2), as well as 27 healthy adults to compare FGF-21 blood concentrations. ELISA was used to assess plasma FGF-21 levels, while body weight, body length, BMI and Z-score were measured immediately after delivery and before discharge from the ward. It was observed that the FGF-21 concentration increases significantly during the first week of life when compared to the concentration measured immediately after birth. Furthermore, FGF-21 values during the first 24 h of life are positively correlated with body weight and length expressed as Z-score, but no correlation was found with BMI Z-score at discharge. Eight of the 25 newborns (32%) had undetectable FGF-21 levels at T1 (they were then assigned a value of 0), but at the T2 measurement FGF-21 concentrations were already detectable and it was observed that, in all babies, FGF-21 concentrations reached higher values at the second measurement. Furthermore, FGF-21 concentrations in all neonates at T2 were significantly higher than adults (471.6 (159.6–786.1) vs. 102.6 (65.8–269.9) pg/mL, *p* = 0.016) [14].

In contrast, Yang et al. [15] analysed whether umbilical cord blood FGF-19 levels are associated with the occurrence of GDM and what effect this has on fetal growth. Potential correlations between sexual dimorphism and cord blood FGF-19 concentrations were also investigated. The study enrolled 153 newborns whose mothers suffered from GDM-the study group, and 153 newborns of healthy mothers without metabolic diseases, i.e., the control group. ELISA assays were used to measure FGF-19 values in the samples. Cord blood plasma levels of FGF-19, insulin, C-peptide, proinsulin, IGF-I and IGF-II were assessed. It was observed that, in the control and study groups regardless of the sex of the baby, cord blood plasma FGF-19 concentrations were not statistically significantly different and were also not correlated with IGF-I or IGF-II values. In neonates born prematurely compared to term born neonates, FGF-19 concentrations reached higher values (64.6 ± 33.4 versus 43.2 ± 28.8 pg/mL, *p* = 0.009) and by caesarean section compared to natural deliveries (47.1 ± 28.7 versus 41.4 ± 29.5 pg/mL, *p* = 0.09). Moreover, FGF-19 concentrations were higher among patients who had undergone elective caesarean section (49.9 ± 30.3 pg/mL, *p* = 0.03) while, if caesarean section was performed due to urgent indications (40.9 ± 24.2 pg/mL, *p* = 0.92) FGF-19 reached similar values compared to natural deliveries (41.4 ± 29.5 pg/mL, *p* = 0.09). FGF-19 concentrations in both groups of female newborns were positively correlated with birth weight (r = 0.23, *p* = 0.01) and body length (r = 0.21, *p* = 0.02), C-peptide (r = 0.27, *p* = 0.002) and proinsulin (r = 0.27, *p* = 0.002). In male newborns, umbilical cord plasma FGF-19 levels were not correlated with birth weight, body length, insulin or proinsulin [15].

The authors of another study analysed serum FGF-23 levels in women with gestational hypertension and in healthy pregnant women and assessed the presence of fetal growth disorders. The control group consisted of 25 healthy pregnant women without co-morbidities whose previous pregnancy had ended in a caesarean section, and 30 patients with gestational hypertension were included in the study group and divided into three subgroups on the basis of hypertension complaints and laboratory results, who also had undergone a caesarean section. A total of 23 patients with pre-eclampsia, gestational hypertension and proteinuria above 300 mg/day were allocated to the first subgroup; the second subgroup consisted of 5 patients with gestational hypertension without proteinuria; and the third subgroup included 2 patients with eclampsia. Furthermore, the entire study group was divided according to estimated fetal weight into a further two subgroups; the first subgroup consisted of 22 patients who were not diagnosed with FGR because their estimated weight was above the 10th percentile, while the second subgroup included 8 patients who were diagnosed with FGR due to an estimated birth weight below the 10th percentile. Serum FGF-23 values were tested with ELISA. The study compared demographic parameters, laboratory results and FGF-23 concentrations, which were similar in both the study and control group. However, it was noted that, on average, delivery occurred at an earlier week in the study group compared to the control group (*p* = 0.002). There was no statistically significant difference in serum FGF-23 concentration between the study group and the control group. In the study group (with and without FGR), serum FGF-23 values in women with gestational hypertension and without FGR were found to be similar to the results in the control group. In contrast, serum FGF-23 and calcium concentrations were significantly lower in patients with gestational hypertension and FGR compared to the control group (*p* = 0.044 and *p* < 0.001). These findings suggest a potential role of FGF-23 and calcium in the pathophysiology of gestational hypertension with co-occurring FGR [16].

The summary of data from presented studies can be seen in Table 1.

## 4. Discussion

In recent years, particular attention has been paid to the role of fibroblast growth factors in maternal and child health. This review examines the role of FGF in fetal growth disorders and related conditions GDM and gestational hypertension. The findings suggest that FGF-21, FGF-19 and FGF-23 may play an important role in the pathogenesis of both fetal hypotrophy and hypertrophy, as well as in metabolic complications associated with pregnancy.

FGFs are important regulators of metabolic processes and their varying concentrations during pregnancy have a potential impact on intrauterine fetal growth and postnatal development.

### 4.1. Summary of Presented Researches

The *Cord blood FGF-21 in GDM and its relationship with postnatal growth* study showed that FGF-21 levels correlate with the BMI-ZS ratio in children of mothers without GDM aged 1 to 4. On this basis, the authors suggest that FGF-21 may act as a biomarker of postnatal growth in children born from normal pregnancies. In contrast, the absence of this correlation in children of mothers with GDM may indicate that fetal hyperglycemia limits the usefulness of FGF-21 as an indicator of postnatal growth in this group [10]. In contrast, Vrachnis et al. [13] noted that higher FGF-21 concentrations in the second trimester of pregnancy were associated with cases of SGA and LGA, highlighting that increased FGF-21 concentrations may have an adaptive effect, increasing energy availability, protecting against oxidative stress and promoting tissue repair, which may be key in cases of FGR.

The Yang MN study was the first to show that GDM does not affect the concentration of FGF-19 in cord blood. In contrast, other studies indicate that the expression of FGF-19 in placenta and muscle tissue is reduced in women with GDM compared to healthy pregnant women [17]. FGF-19 deficiency can lead to abnormalities in bile acid metabolism, which negatively affects the regulation of glucose–lipid metabolism, exacerbating the insulin resistance characteristic of GDM. Consequently, this may increase the risk of metabolic disorders in both the mother and the developing fetus [17,18].

Whereas a study by Joung et al. [13] provides important information on the role of FGF-21 in the growth of preterm infants, it should be noted that, despite significant correlations between FGF-21 concentrations and body weight and length, no association was shown with BMI Z-score. This may indicate that FGF-21 plays a more complex role in the growth process, perhaps affecting muscle and bone mass more than fat tissue gain. Such an observation suggests that the role of FGF-21 in the context of preterm deliveries requires further research to explain its full mechanism [13].

Furthermore, analysis of the changes in FGF-19 and FGF-21 concentrations in early infancy in a study by Sánchez-Infantes et al. [12] showed that the values were low at birth irrespective of prenatal growth and increased significantly in the first year of life. This phenomenon may be related to metabolic adaptation and intensive neonatal growth. Furthermore, it was noted that the proteins of the FGF-19 subfamily had different rates of increase—FGF-19 concentration in blood grew slower than FGF-21. The authors suggest that the delayed increase in FGF-19 levels may be due to its synthesis in the intestine, which is still immature at birth and requires postnatal development to reach full function. Additionally, the study highlighted that the increase in FGF-21 values was very similar in AGA and SGA infants, while the increase in FGF-19 was reduced in SGA infants [12].

An interesting aspect is that of gender-based differences in cord blood FGF-19 and FGF-21 concentrations. In the study of Yang et al. [15], only in the group of female newborns did FGF-19 levels positively correlate with birth weight and body length. The researchers suggest that the positive correlation between FGF-19 and the birth weight of female newborns may be due to the dimorphic role of this factor in fetal growth and development. This indicates that there may be gender-specific mechanisms that affect metabolic regulation during the prenatal period. It is known that female neonates show greater insulin resistance than male neonates, and differences in fetal growth may be determined by different intrauterine hormonal and metabolic factors [19,20]. These observations require further research to confirm the new findings and explain the underlying mechanisms. In the study by Megia et al. [10], it was shown that females born to mothers with GDM had higher cord blood FGF-21 levels compared to males. Such differences may be significant in terms of children’s later metabolic health, which requires further investigation. This dimorphism may be due to differences in the hormonal and metabolic maternal environment and the influence of genetic and epigenetic factors [10,15].

According to the *Evaluation of maternal serum fibroblast growth factor-23 levels in FGR and gestational hypertensive disease study*, FGF-23 plays an important role in the pathogenesis of gestational hypertension and fetal growth disorders. The lower levels of FGF-23 and calcium in patients with FGR and gestational hypertension suggest a possible dysfunction of the electrolyte axis, which may contribute to fetal growth abnormalities. What is more, in the study of Qamar et al. [21], it was observed that higher maternal blood levels of FGF-23 reduced the risk of SGA. These findings highlight that FGF-23 may be a biomarker of fetal growth, particularly relevant in complicated pregnancies [16].

It is necessary to highlight that fetus weight under the 10th centile does not meet the full definition of FGR. Karacan et al. [16] divided participants according to estimated fetal weight into a further two subgroups; the first subgroup were not diagnosed with FGR because estimated weight was above the 10th percentile, while the second subgroup had estimated birth weight below the 10th percentile. In some cases, the rate of growth may be insufficient, despite estimated fetal weight exceeding 10th centile. Therefore, during FGR diagnosis, it is also recommended to analyze Doppler parameters in the umbilical artery: an increased pulsatility index or the presence of reversed/zero flow in the diastolic phase suggests placental insufficiency, and a decrease of ≥2 percentiles in subsequent ultrasound examinations indicates growth retardation and comparison of the dynamics of abdominal circumference growth in relation to head circumference [22]. The inclusion of these criteria allows for more precise detection of FGR cases and reduces the risk of misclassification of fetuses. In future studies and clinical practice, it is worth using an integrated approach to more precisely characterize the patho-mechanism of fetal growth disorders.

### 4.2. Summary of FGF-19 Subfamily

In summary, FGF-21 appears to be a key marker and regulator of both intrauterine and early postnatal metabolic adaptation. Sánchez-Infantes et al. [12] showed that FGF-21 levels increase rapidly in the first week of life—exceeding values observed in adults—and remain elevated for the first year among infants. This suggests an adaptive function of FGF-21 in response to increased energy requirements after birth. What is more, Vrachnis et al. [13] confirmed that both SGA and LGA fetuses have higher FGF-21 levels in amniotic fluid than AGA fetuses, suggesting a compensatory mechanisms in the setting of disrupted growth patterns. Furthermore, female neonates born to mothers with GDM had significantly higher FGF-21 level than males, suggesting stronger early metabolic signaling among girls [10]. To sum up, these results indicate that FGF-21 is not only an indicator of metabolic adaptation in the perinatal period but also an active growth mediator, which effects differently depending on the sex of the child.

The mechanisms of FGF-19 action in fetal development are not yet fully understood, but recent studies indicate its role in postnatal metabolic adaptation and the effect of sexual dimorphism. Sánchez-Infantes et al. [12] showed that, in all newborns, regardless of birth weight, FGF-19 concentrations in cord blood are initially low and then increase rapidly until the fourth month of life. However, among SGA infants, FGF-19 increase was much weaker compared to AGA children, suggesting impaired metabolic adaptation after birth [12]. In addition, Yang et al. [15] noted that, among females, FGF-19 concentration in cord blood was positively correlated with birth weight, body length, C-peptide and proinsulin levels, whereas such correlations were not observed among males. These differences indicate that FGF-19 may affect fetal growth in a sex-dependent differences, although the pathways of this action require further research.

The role of FGF-23 in perinatal and early infant growth has received the least attention. In the only study included in our review, no overall differences in FGF-23 levels were found, when comparing women with gestational hypertension with healthy pregnant women [16]. However, patients, who were additionally diagnosed with FGR, had significantly lower levels of both FGF-23 and calcium [16]. These results support the hypothesis that FGF-23 is involved in the pathophysiology of GDM and FGR. Due to limited data, further detailed studies are needed to clarify how FGF-23 interacts with the calcium and phosphate metabolism in the prenatal period and how it impacts fetal development.

### 4.3. Strengths and Limitations

The main strength of the presented review is focusing only on the FGF-19 subfamily, which allows examination in detail on its role in prenatal and neonatal growth. In addition, each member of the FGF-19 subfamily was analysed individually in order to have clearer insights into its physiological significance and clinical correlations. The review also highlights sex-specific differences, perinatal issues, including preterm birth, SGA, LGA, and delivery mode, and the presence of diseases such as GDM and gestational hypertension, which enable a particular interpretation of FGFs in the broader framework of maternal–fetal health and increase the validity and relevance of the findings.

It is also necessary not to ignore the limitations of the presented studies. First of all, only six original papers were included in this review due to the inclusion and exclusion criteria, and in each study the number of participants in the control and study groups was quite small, which may limit the generalizability of the conclusions presented. What is more, all the described studies had a different methodology, timing of collecting samples from patients, samples sizes, and ELISA kits, which is why a meta-analysis was not conducted, and exact comparisons across studies are, therefore, more complicated.

### 4.4. New Directions

Currently, there are many research projects expanding knowledge of the FGF’-19 subfamily. Especially, FGF-21 is being studied in the pathophysiology of GDM. Recently, Sun et al. [23] examined the correlation of FGF-21 and L-cystine in pregnant women with GDM. The researchers found that patients with GDM have increased L-cystine in the blood and a positive correlation between L-cystine and FGF21 levels. Moreover, the authors proved that L-cystine increases the expression of the antioxidant NRF-2 pathway activated by FGF-21, which has protective effect on endothelial cells damaged by high glucose concentrations in GDM. These findings suggest that FGF21 is a potential therapeutic agent for GDM, especially in the protection of endothelial cells. Nevertheless, Caltek et al. [24] studied the level of FGF-21 in pregnant women with GDM and healthy patients. It was confirmed once again that women with GDM have higher concentrations of FGF-21. Participants with increased AFI also had higher concentrations of FGF-21 than women with AFI within the normal range. These findings confirms the role of FGF-21 in the regulation of maternal glycemia and suggests its potential as a noninvasive biomarker of GDM.

What is more, the FGF-19 subfamily is not only studied in perinatology. Recently, the levels of FGF-19 and FGF-21 were evaluated among patients after liver transplantation along with their correlations with liver function markers and inflammatory parameters. It was observed that FGF-19 reached the highest concentrations before liver transplantation, then 24 h after transplantation decreased significantly, and within two weeks its values increased. In contrast, FGF-21 reached the lowest concentration before transplantation, the maximum 24 h after transplantation, and then declined to initial values within two weeks. Moreover, fluctuations in the levels of FGF-19 and FGF-21 correlated with changes in levels of liver enzymes: alanine transaminase (ALT), aspartate transaminase (AST), gamma-glutamyl transferase (GGT), alkaline phosphatase (ALP) and C-reactive protein (CRP), indicating that these FGFs respond to the liver injury and recovery phases. In conclusion, the changes in FGF-19 and FGF-21 levels in blood reflect their potential roles among patients after transplantation/ FGF-19 may have a protective effect on the graft during the period of ischemia and subsequent reperfusion, whereas FGF-21 seems to enhances hepatocyte regeneration. Monitoring hormone concentrations may provide new insights into the function of the transplanted liver, highlighting their potential as biomarkers for transplant monitoring [25]. In addition, a phase 2b controlled trial is currently underway for the use of Pegasofermin—FGF-21 recombinant analogue for patients with histo-pathologically confirmed non-alcoholic steatohepatitis (NASH) and liver fibrosis. Researchers report that Pegasofermin influences the pathophysiology of the disease and thus inhibits processes contributing to fibrosis [26].

## 5. Conclusions

In conclusion, the data indicate complex correlations between FGF-19 subfamily factors and fetal and neonatal growth. Differences in FGF-19 and FGF-21 concentrations based on gender and gestational disorders suggest the need for further research in order to fully understand the effects of these proteins and their potential clinical applications. All the studies discussed suggest that FGF-21, FGF-19 and FGF-23 may play an important role in fetal and neonatal growth and development, particularly in pregnancies complicated by metabolic disorders, such as GDM or gestational hypertension.

## Figures and Tables

**Table 1:** The comparison of the role of FGF-19, FGF-21 and FGF-23 in fetal and neonatal growth [10,12,13,14,15,16].

Title of the Study:	Participants	FGF Type	Type of Sample	The Results
*Cord blood FGF21 in gestational diabetes and its relationship with postnatal growth*	157 pregnant women, including 79 GDM 78 healthy women, and their newborns	FGF-21	maternal plasma blood cord blood	higher concentration of FGF-21 among patients with GDM compared to healthy pregnant women. no significant differences in FGF-21 concentrations in cord blood in the children of patients from either group females born to women with GDM had higher cord blood FGF-21 concentrations compared to males
*Circulating FGF19 and FGF21 surge in early infancy from infra- to supra-adult concentrations*	22 AGA infants, 22 SGA infants; 35 healthy adults	FGF-19 FGF-21	serum samples collected: at birth (cord blood), 4 and 12 months serum samples collected from healthy adults	The FGF-21 concentration: at birth were low, increased significantly during the first week of life and remained at a higher level during the first year- in AGA and SGA groups; among AGA group, FGF-19 concentration peaked at 4 months then declined; SGA infants had lower level of FGF-19 at 4 months than AGA children
*Increased Fibroblast Growth Factor 21 (FGF21) Concentration in Early Second Trimester Amniotic Fluid and Its Association with Fetal Growth*	80 fetuses: 31 SGA, 18 LGA, 31 AGA	FGF-21	amniotic fluid	higher FGF-21 concentration in SGA and LGA-association with abnormal fetal growth maternal age, female sex of the fetus and smoker status of the mother were only associated with SGA
*Association of circulating FGF-21 levels in the first week of life and postnatal growth in hospitalized preterm infants*	25 preterm neonates 27 healthy adults	FGF-21	blood samples were collected at two time points: within 24 h of life (T1), and 24–96 h after the first blood draw (T2)	the FGF-21 concentration increases significantly during the first week of life the FGF-21 values during the first 24 h of life are positively correlated with body weight and length expressed as Z-score, but no correlation was found with BMI Z-score at discharge. the FGF-21 concentrations in all neonates at T2 were significantly higher than in adults
*Fibroblast Growth Factor 19 in Gestational Diabetes Mellitus and Fetal Growth*	306 infants (153 both to mothers with GDM, 153 born to healthy mothers)	FGF-19	cord blood	in the control and study groups regardless of the sex of the baby, cord blood plasma FGF-19 concentrations were not statistically significantly different in neonates born prematurely compared to term born neonates, FGF-19 concentrations reached higher values, and by caesarean section compared to natural deliveries the FGF-19 concentrations positively correlated with BW, length, proinsulin only in females, in both groups
*Evaluation of maternal serum fibroblast growth factor-23 levels in fetal growth restriction and gestational hypertensive disease*	30 patients with gestational hypertension (23 pre-eclampsia, 5 gestational hypertension, 2 eclampsia); 25 healthy pregnant women	FGF-23	maternal serum	no statistically significant difference in serum FGF-23 concentration between the study group and the control group. serum FGF-23 and calcium concentrations were significantly lower in patients with gestational hypertension and FGR compared to the control group

## Data Availability

Data derived from public domain resources.
